# Supplementary material for: Reinforcing Gaps? A Rapid Review of Innovation in Borderline Personality Disorder (BPD) Treatment
Source: Brain Sci. 2025 Jul 31;15(8):827. doi: 10.3390/brainsci15080827 (PMC12384757; doi:10.3390/brainsci15080827)
Supplement: Supplementary file 1 [file brainsci-15-00827-s001.zip › bpd_extracted_articles_.pdf]

| First author | Year | graphic set | tudy desig | nary objec | lusion crite | lusion crite | mple size ( | ean age (± | % female |
|---|---|---|---|---|---|---|---|---|---|
| Vonderlin | 2025 | Germany | Interventio | To assess a | Diagnosis o | None repo | 39 (ITT); 18 | 31.1 (10.6) | 76.9% |
| Neuner | 2024 | Germany | Randomize | To evaluat | Diagnosis o | Acute psyc | 58 (29 NET | 33.8 (9.4) | 81.0% |
| Jones | 2023 | UK | Randomize | To assess t | Severe BPD | NR | 80 | 35.2 (11.0) | 70% |
| Schneider | 2024 | Germany | Pragmatic, | To evaluat | BPD diagno | NR | 120 | 29.4 (8.2) | 78% |
| Smith | 2022 | USA | Randomize | To compar | BPD diagno | NR | 50 | 32.0 (9.5) | 65% |
| Lee | 2025 | Australia | Nonrandor | To test effe | BPD diagno | NR | 45 | 30.5 (10.1) | 80% |
| Müller | 2023 | Germany | Randomize | To test clin | BPD diagno | Neuromus | 30 | 34.7 (12.3) | 60% |
| Garcia | 2024 | USA | Pilot study | To add EM | BPD and P | NR | 20 | 29.0 (8.8) | 85% |
| Becker | 2023 | Germany | Observatio | To examin | BPD diagno | NR | 25 | 33.2 (11.5) | 68% |
| Rossi | 2025 | Italy | Randomize | To evaluat | BPD diagno | NR | 40 | 30.1 (9.7) | 75% |
| Rothman | 2024 | USA | Feasibility | To evaluat | BPD diagno | None repo | 25 | 28.5 (6.7) | 88% |
| Bartsch | 2024 | Germany | Double-bli | To investig | DSM-5 BPD | Pregnancy, | 60 | 30.9 (8.2) | 77% |
| Hansen | 2023 | Denmark | Randomize | To evaluat | BPD diagno | Severe sub | 84 | 31.2 (9.8) | 72% |
| Dwyer | 2023 | UK | Open-label | To assess f | BPD diagno | Neurologic | 15 | 29.8 (6.5) | 80% |
| Fineberg | 2024 | UK | Randomize | To evaluat | BPD; mooc | Bipolar dis | 100 | 33.5 (10.1) | 67% |
| Assmann | 2025 | Germany | Pragmatic, | To test the | Aged ≥18; | Primary su | 580 (302 ir | 29 (IQR 24 | 90% (520/5 |
| Bozzatello | 2023 | Italy | Randomize | To evaluat | Age 18–60 | Dementia | 46 (24 IPT- | NR | NR |
| Alavi | 2021 | Canada | Nonrandor | To evaluat | Age 18–65 | Acute hypo | 107 (52 e-I | NR | NR |
| Wollmer | 2022 | Germany | Single-blin | This 16-we | Female; ag | Psychiatric | 53 (27 BTX | BTX: 30.44 | 100% |
| Wilhelmus | 2023 | The Nethe | Non-concu | To examin | Age >18; D | IQ <75; ins | 12 (9 comp | 30.6 (12.4) | 83% |
| Schulze | 2024 | Germany | Seed-based | To present | Women; ag | Not specifi | 45 (24 BTX | BTX: 28.75 | 100% |
| Chanen | 2021 | Australia | Single-blin | To evaluat | Age 15–25 | None repo | 139 randor | 19.1 (2.8) | 81.3% |
| Herpertz | 2020 | Germany | Cluster-rar | To evaluat | DSM-IV BP | Not detaile | 59 | NR | NR |
| Quattrini | 2024 | Italy | Randomize | To investig | DSM-IV TR | Schizophre | 47 | NR | NR |
| Dwyer | 2025 | 17 countrie | Phase 2 ra | To provide | Age 18–65 | Other prim | 390 randor | 30.2 (10.3) | 86.2% |
| Fineberg | 2023 | USA | Double-bli | To collect i | Age 21–60 | Psychotic c | 22 random | Ketamine 3 | Ketamine 8 |
| Klein | 2021 | Germany | Randomize | To determ | DSM-IV BP | Psychotic c | 204 randor | 32.4 (9.7) | 91.7% |
| Schindler | 2024 | Germany | Prospectiv | To compar | ICD-10 BPD | Organic me | 100 (50 ICD | NR | NR |
| Hurtado-Sa | 2022 | Spain | Preliminary | To test the | Age 15–30 | Antisocial I | 40 (20 IT; 2 | 20.53 (4.32 | 82.5% |
| Salvatore | 2021 | Italy | Single case | To illustrat | Single youn | NR | 1 | NR | 100% |
| Mohajerin | 2025 | Iran | Randomize | To compar | Age 11–17 | Schizophre | 91 (46 UP-A | UP-A: 15.9 | UP-A: 65.2 |
| Kleindienst | 2021 | Germany | Subgroup a | To compar | Women; D | Schizophre | 93 women | Not specifi | 100% |
| Moran | 2024 | UK | Feasibility | To assess f | Age ≥18; D | Psychotic c | 48 (n=24 E | Adults: 32. | 100% |
| Kujovic | 2024 | Germany | Single-blin | To evaluat | Female; 18 | Psychotic c | 53 random | BTX: 30.44 | 100% |
| Soler | 2022 | Spain | Feasibility | To examin | 18–65; DSI | Life-threat | 32 particip | Not report | Not report |
| Laursen | 2021 | Denmark | Pragmatic | To perform | ≥18; ICD-1 | No smartp | 78 random | 29 (not spe | 86.2% |
| Harty | 2024 | The Nethe | Case repor | To offer ins | Woman wi | NR | 1 | NR | 100% |
| Francis | 2024 | Malaysia | Case series | To highligh | BPD patier | NR | 3 cases | NR | 100% |
| Schmeck | 2022 | Switzerland | Nonrandor | To demons | Age 13–19 | IQ<80; psy | 60 (37 DBT | NR | NR |
| Fitzpatrick | 2025 | Canada (To | Uncontroll | To present | DSM-5 BPD | Severe IPV | 16 couples | Not report | Not report |
| Molavi | 2020 | Iran | Randomize | To investig | DSM-IV & I | Dominant | 32 (16 acti | 30.63 (5.39 | 100% |
| Sosic-Vasic | 2024 | Germany | Two-sessio | To evaluat | Female; DS | Psychotic c | 48 (24 IR; 2 | Not report | 100% |
| Back | 2022 | Germany | Randomize | Reduced va | Unmedicat | Neurologic | 113 (53 BP | BPD: 30.02 | 100% |
| Juul | 2022 | Denmark | Case repor | A clinical il | Young wor | NR | 1 | 28 | 100% |
| Calderón-M | 2020 | Mexico | Double-bli | Improvem | DSM-IV BP | Contraindi | 14 (active v | 26.0 (7.08) | 78.6% |
| Danayan | 2023 | Canada | Retrospect | To evaluat | Adults with | NR | 100 (50 BP | BPD+: ?, B | NR |

| | | | | | | | | | |
|---|---|---|---|---|---|---|---|---|---|
| Vanicek | 2022 | Austria | Case repor | To report c | Adult fema | NR | 1 | 20 | 100% |
| Rossi | 2023 | Italy | Randomize | To assess c | Adults 18– | Psychotic, | 78 (39 MIT | NR | NR |
| Guillén | 2024 | Spain | Randomize | To carry ou | Age >18; re | Primary su | 121 relativ | NR | NR |
| Hood | 2024 | USA | Randomize | To determ | Age ≥18; D | Schizophre | 84 trauma- | 28.49 (9.18 | 74.4% |
| Hafkemeije | 2023 | Netherland | Case repor | To evaluat | DSM-5 BPD | PTSD diagr | 2 | NR | 100% |
| Bo | 2022 | Denmark | Case repor | To illustrat | 16-year-old | NR | 1 | 16 (NR) | 100% |
| Bozzatello | 2022 | Italy | Randomize | To evaluat | DSM-5 BPD | Delirium; c | 43 (22 IPT- | NR | NR |
| Vaz | 2020 | Portugal | Case repor | To demons | Adult with | NR | 1 | NR | 100% |
| Mohajerin, | Iran | Randomize | To compar | Adults ≥18 | Substance | 55 MBT; 53 | NR | NR | Psychother |
| Tinlin-Dixo | UK | Case repor | Using Cogr | 68-year-old | NR | 1 | 68 | 100% | Psychother |
| Dunand; 20 | Switzerlan | Multiple ca | To explore | Age >18; re | Primary su | 6 | 32.2 | 83.3% | Other (Fam |

| tion classif | rvention n | n (weeks/r | Frequency | Format | dules/com | parator/Co | rtality (Y/ | ity/mortali | symptoms |
|---|---|---|---|---|---|---|---|---|---|
| Psychother | Online Dial | 12 months | Weekly mo | Individual, | Mindfulnes | None | No | No | None repo |
| Psychother | Narrative E | 4 weeks | 8 sessions | Individual | Trauma na | Treatment | No | No | PTSD-relat |
| Pharmacol | Clozapine | 12 weeks | Daily dosin | Individual | NR | Placebo | Yes; numb | Yes; advers | Sedation, v |
| Digital tool | EPADIP-BP | 8 weeks | Daily use | Self-guided | DBT-based | TAU | NR | NR | NR |
| Psychother | Group Inte | 16 weeks | Weekly gro | Group | Interperso | DBT skills g | NR | NR | NR |
| Digital tool | Email-base | 6 weeks | Twice wee | Individual | DBT skills v | Email psyc | NR | NR | NR |
| Neuromod | Glabellar B | 4 weeks fo | Single injec | Clinical pro | Botulinum | Saline injec | NR | Yes; injecti | Headache; |
| Psychother | EMDR add | 8 weeks | Weekly ses | Individual | EMDR prot | Standard B | NR | NR | NR |
| Neuromod | Glabellar B | Pre-post 2 | Single injec | Clinical & i | Resting-sta | No-treatm | NR | NR | NR |
| Psychother | Metacogni | 12 weeks | Weekly ses | Individual | Metacogni | Supportive | NR | NR | NR |
| Digital tool | DBT Coach | 4 weeks | Daily use | Individual | Emotion re | None | NR | NR | NR |
| Pharmacol | Intranasal | 4 weeks | Twice daily | Individual | Oxytocin 2 | Placebo | NR | NR | None repo |
| Psychother | Brief Ment | 12 weeks | Weekly | Group | Mentalizat | Treatment | NR | NR | NR |
| Digital tool | VR Emotio | 4 weeks | Twice wee | Individual | Emotion re | None | NR | NR | NR |
| Pharmacol | Lamotrigin | 12 weeks | Daily | Individual | Titrated la | Placebo | NR | Rash repor | Headache; |
| Digital tool | Priovi digit | 12 months | Recommen | Unguided | Schema–m | Treatment | Yes; fewer | No differen | None repo |
| Psychother | Individual | 20 weeks | Weekly ses | Individual | Clarificatio | Individual | No | NR | NR |
| Digital tool | Email-base | 15 weeks | Weekly | Email hom | Mindfulnes | In-person | NR | NR | NR |
| Neuromod | Glabellar B | Single injec | One-time | Clinical pro | 34U incobo | Minimal ac | No | Yes; mild A | Headache; |
| Psychother | Eye Mover | 15 weeks T | Weekly TA | Individual | Dutch EMD | Within-sub | No adverse | No | Not report |
| Neuromod | Glabellar B | Single injec | One-time | Clinical & i | 34U BTX in | Minimal ac | Not report | Not report | Not report |
| Psychother | HYPE + CA | 12 months | Weekly CA | Individual | CAT relatio | Active com | No | No | None repo |
| Psychother | Mechanism | 6 weeks | Two 1.5-ho | Group | DBT and M | Non-specif | No | No | None repo |
| Psychother | Metacogni | 1 year; we | Weekly inc | Individual | Metacogni | Structured | Not report | Not report | Not report |
| Pharmacol | BI 135889 | 12 weeks | Once daily | Oral medic | Flexible tit | Placebo | No increas | Yes; AEs 77 | Headache; |
| Pharmacol | Ketamine ( | Single infus | One-time | IV infusion | Antidepres | Midazolam | No serious | Transient c | Dissociativ |
| Digital tool | Priovi inter | 12 months | Recommen | Unguided | Schema th | Care as usu | No deaths; | No SAEs di | None repo |
| Psychother | Integrated | 12 months | Ongoing D | Team-base | DBT routin | Treatment | No deaths | Serious adv | NR |
| Psychother | Iconic Ther | 10 weeks i | Weekly gro | Group + in | Image-base | Supportive | No deaths | No SAEs | NR |
| Psychother | MIT | Not specifi | Not specifi | Individual | Schema fo | None | NR | NR | NR |
| Psychother | Unified Pro | 12 months | UP-A: wee | Individual | UP-A: emo | Active com | No deaths | No | None repo |
| Psychother | DBT-PTSD; | 12 months | Weekly ses | Individual | DBT skills + | CPT | No deaths | No differen | None repo |
| Digital tool | Perinatal E | 12 weeks f | Weekly 2-h | Online gro | Mindfulnes | Treatment | No deaths | No serious | None repo |
| Neuromod | Intermitter | 8 weeks | Daily M–F, | Clinical pro | 600 pulses | Sham stim | No | Yes; mild a | Headache; |
| Psychother | Expanded | 12 weekly | Weekly gro | Group | Self-care (F | None | No | No | None repo |
| Digital tool | mDiary mo | 12 months | Daily entri | Digital adju | Emotion, u | Paper-base | No deaths | No serious | None repo |
| Psychother | Bi-monthly | 2 months | Bi-monthly | Individual | Embodied | None | NR | NR | NR |
| Pharmacol | Brexpipraz | Up to 12 w | Once daily | Oral medic | D2/D3 par | None | Patients ha | Yes; some | Akathisia, i |
| Psychother | Adolescent | 6–8 month | 25 weekly | Individual | TFP techni | Active com | No deaths | No differen | NR |
| Psychother | Sage conjo | 12 sessions | Weekly | Dyadic con | Phase 1 sa | None (sing | No deaths | No serious | None repo |
| Neuromod | Repeated | 10 days | 10 sessions | Single-site | Anodal left | Sham stim | No serious | Yes; mild A | Headache; |
| Psychother | Two-sessic | Two sessio | Weekly ses | Individual | Rescripted | Treatment | No deaths | No SAEs | None repo |
| Pharmacol | Intranasal | Single adm | One-time | Clinical tria | 24 IU oxyto | Placebo sp | No (NR) | No (NR) | Not report |
| Psychother | Short-term | 20 weeks | Weekly gro | Group + in | MBT-I intro | None | NR | NR | NR |
| Neuromod | 5 Hz rTMS | 3 weeks (1 | 5 sessions/ | Clinical pro | 30 trains × | Sham rTMS | No | No serious | Headache |
| Pharmacol | IV Ketamin | 2 weeks | Four infusi | Clinical out | Flexible do | Matched T | No deaths; | Yes; dissoc | Dissociatio |

| | | | | | | | | | |
|---|---|---|---|---|---|---|---|---|---|
| Pharmacol | IV Esketam | 2 weeks | Five infusic | Inpatient c | Adjunct to | None | No deaths; | No serious | Disinhibitic |
| Psychothe | Metacogni | 12 months | Weekly inc | Mixed indi | Metacogni | Structured | No | No | NR |
| Other (Fan | Family Cor | 12 weeks ( | Weekly 2-h | Group | Psychoedu | Treatment | No | No | None repo |
| Psychothe | BPD Comp | 18 weeks | Weekly 50 | Individual | Values ide | Waitlist co | No | No | None repo |
| Psychothe | Intensive E | 4 consecut | 10 session | Individual | Processing | None | No | No | None repo |
| Psychothe | Socioecolo | Not specifi | Not specifi | Individual | Mentalizat | None | NR | NR | None repo |
| Psychothe | Interperso | 10 months | Weekly 50 | Individual | Symptom r | Waiting lis | No deaths | No serious | Headache |
| Psychothe | Therapeuti | 1 year ther | Varied ses | Individual | Reactivatic | None | No | No | None repo |
| Name: Me | Active com | Suicide mc | SCID-5-PD | None reported | | | | | |
| Name: Cog | None | Suicide mc | PSQ = Pers | Elderly (>65) | | | | | |
| Name: Fan | Treatment | Suicide mc | BAS = Burc | None reported | | | | | |

| ptoms (Y/N | iatric disor | l functiona | l impact (c | (acronyms | populations included |
|---|---|---|---|---|---|
| Yes; BSL-2 | Dissociativ | Quality of | NR | BSL-23; DS | None reported |
| Yes; meas | PTSD symp | Significant | NR | BPDSI; CAF | None reported |
| Yes; meas | Psychotic s | GAF | NR | BPDSI; GAI | NR |
| Yes; BSL-2 | NR | WHOQOL- | NR | BSL-23; WI | NR |
| Yes; BPDSI | NR | SAS-SR | NR | BPDSI; SAS | NR |
| Yes; BSL-2 | NR | WHO-5 | NR | BSL-23; WI | NR |
| Yes; BPDSI | NR | NR | NR | BPDSI | NR |
| Yes; BSL-2 | PTSD symp | NR | NR | BSL-23; PC | NR |
| NR | NR | NR | NR | fMRI conn | NR |
| Yes; BPDSI | NR | NR | NR | BPDSI; DTI | NR |
| Yes; BSL-2 | NR | NR | NR | BSL-23 | None reported |
| Yes; ZAN-B | Depressive | NR | NR | ZAN-BPD | None reported |
| Yes; BPDSI | Depressior | Improved | NR | BPDSI; WH | None reported |
| Yes; BSL-2 | NR | NR | NR | BSL-23 | None reported |
| Yes; BPDSI | Affective ir | NR | NR | BPDSI | None reported |
| Yes; BSL-2 | Anxiety (G | SF-12 men | None repo | BSL-23 = B | Gender diverse included (2%). |
| Yes; BPDSI | Aggression | SOFAS; SA | NR | BPDSI; CGI | None reported |
| Yes; DERS | NR | NR | NR | SAQ = Self- | None reported |
| Yes; ZAN-B | Depressior | Not report | Not report | ZAN-BPD = | None reported |
| Not report | PTSD (PCL- | Disability v | Not report | PCL-5; KKL | None reported |
| Yes; BSL-2 | Not report | Not report | Not report | rsFC; M1; | None reported |
| Yes; psych | Depressive | IIP Circum | Treatment | IIP-C = Inve | Young people (15–25) |
| Yes; aggres | Not report | Not report | Not report | M-OAS = M | None reported |
| Yes; clinica | Not report | Not report | Not report | FA = fracti | None reported |
| Yes; ZAN-B | Depressior | Not report | Not report | ZAN-BPD; | None reported |
| Not report | Depressior | Improved | Not report | BSS; BDI; B | None reported |
| Yes; BPDSI | Not report | Not report | Not report | BPDSI; SAE | None reported |
| Yes; BSL-2 | PHQ-9; GA | GAF impro | Hospital da | ICB; DBT; | None reported |
| Yes; BSL-2 | Suicidal ide | Maladjustr | NR | IT; SI; BSL- | Youth (15–30) |
| Yes; clinica | NR | Improved i | NR | MIT; CCRT | None reported |
| Yes; BSL-2 | Impulsivity | Not report | Not report | BSL-23; SC | None reported |
| Yes; BSL in | PTSD symp | GAF | Not report | ZAN-BPD; | None reported |
| Yes; BSL-2 | Emotional | WSAS favo | Interventic | BSL-23; CO | Pregnant and postpartum individuals |
| Yes; ZAN-B | Depressior | Not report | Not report | ZAN-BPD; I | None reported |
| Yes; qualit | None repo | Improved | Retention | DBT-ST; W | None reported |
| Yes; reduc | Depressior | QALY gain; | Cost differ | EQ-5D-5L; | None reported |
| Yes; impro | PTSD | Improvem | NR | SIFTing; En | None reported |
| Yes; impro | Cyclothym | Improved | NR | ZAN-BPD; | None reported |
| Yes; BPD c | Depressior | Significant | NR | C-GAS; CIS | Adolescents |
| Yes; BSL-2 | PTSD in FI | QoL meas | Not report | BSL-23 = B | None reported |
| Yes; impro | Depressior | Not report | Not report | ESQ; ERQ; | None reported |
| Yes; reduc | Depressior | Improved i | Not report | BSL-23; SA | None reported |
| Yes; BPD v | PTSD, MDI | Not report | Not report | RMSSD = r | None reported |
| Yes; clinica | Complex P | Improved | NR | MBT = Me | None reported |
| Yes; CGI-B | Depressior | Decision-m | Not report | CGI-BPD; B | None reported |
| Yes; BSL-2 | Depressior | Functional | NR | QIDS-SR16 | NR |

| | | | | | |
|---|---|---|---|---|---|
| Yes; impuls | MDD, eatir | NR | NR | NR | None reported |
| Yes; ZAN-B | NR | IIP, GAF im | NR | DERS; ZAN | None reported |
| Yes; impro | Depressior | Family emp | None repo | BAS = Burc | None reported |
| Yes; ZAN-B | PTSD (PCL- | Not report | Not report | ZAN-BPD = | None reported |
| Yes; no lon | PTSD exclu | Improved ( | Not report | CAPS-5; SC | None reported |
| Yes; clinica | NR | Improved s | NR | Epistemic t | Adolescent |
| Yes; reduct | PTSD, depr | GAF impro | NR | CGI-S; BPD | Adults |
| Yes; disapp | Complex tr | Improved f | NR | BSL-23; TR | None reported |
