# Supplementary material for: Reinforcing Gaps? A Rapid Review of Innovation in Borderline Personality Disorder (BPD) Treatment
Source: Brain Sci. 2025 Jul 31;15(8):827. doi: 10.3390/brainsci15080827 (PMC12384757; doi:10.3390/brainsci15080827)
Supplement: Supplementary file 1 [file brainsci-15-00827-s001.zip › Concepts - supplemental.pdf]

Borderline personality disorder	Therapeutic innovations	Studies/clinical trials
**MeSH**<br><br>"Borderline Personality Disorder"[Mesh]<br><br>**Keywords (title, abstract)**<br><br>Borderline personality(ies)<br>Borderline state(s)	**MeSH**<br><br>"Therapeutics"[Mesh]<br>"Psychotherapy"[Mesh]<br>"Drug Therapy"[Mesh]<br>"Pharmacology"[Mesh]<br>"Internet-Based Intervention"[Mesh]<br>"Digital Health"[Mesh]<br>"Mobile Applications"[Mesh]<br>"Suicide Prevention"[Mesh]<br>"Suicidal Ideation"[Mesh]<br>"Mortality"[Mesh]<br>"Psychosocial Functioning"[Mesh]<br>"Comorbidity"[Mesh]<br>"Recurrence"[Mesh:NoExp]<br>"Mental Health Recovery"[Mesh]<br>"Social Stigma"[Mesh]<br>"Interpersonal Relations"[Mesh]<br>"Cost of Illness"[Mesh]<br>"Quality of Life"[Mesh]<br><br>**Keywords (title, abstract)**<br><br>Innovation(s)<br>Novel<br>Treatment(s)<br>Intervention(s)<br>Therapy(ies)<br>Therapeutic<br>Psychotherapy(ies)<br>Psychotherapeutic<br>Pharmacology (ical)<br>Psychopharmacology (ical)<br>Medication(s)<br>Digital<br>Online/on-line<br>E-mental<br>Internet-based	**MeSH**<br><br>"Clinical Study" [Publication Type]<br><br>**Keywords (title, abstract)**<br><br>** Recherche adjacence*<br><br>Clinical trial(s)<br>Clinical study(ies)<br>Randomized-control trial(s)<br>Observational study(ies)<br><br>OR<br><br>*Available filters in the database*

	Web-based<br>Mobile app(s)/application(s)<br>Neuromodulation<br>Microbiome-based<br>Suicide prevention<br>Preventing suicide(s)<br>Suicidal ideation(s)<br>Mortality<br>Physical health<br>Symptom(s)<br>symptomatology<br>Functional impairment(s)<br>functioning<br>Comorbid<br>Comorbidity<br>Co-occurring<br>Relapse<br>Quality of life<br>Well-being<br>Wellness<br>Recovery<br>Remission<br>Societal impact(s)<br>Societal burden(s)<br>Interpersonal<br>Stigma<br>Stigmatization	

Limits: 2020-2025

English, French

Filters: Clinical trials, clinical studies, observational studies

Concept 1

"Borderline Personality Disorder"[Mesh] OR "borderline personality"[TIAB:~3] OR "borderline personalities"[TIAB:~3] OR "borderline state"[TIAB:~3] OR "borderline states"[TIAB:~3]

Concept 2

"Clinical Study" [Publication Type] OR "clinical trial"[TIAB:~3] OR "clinical trials"[TIAB:~3] OR "clinical study"[TIAB:~3] OR "clinical studies"[TIAB:~3] OR

"randomized trial"[TIAB:~4] OR "randomized trials"[TIAB:~4] OR "observational study"[TIAB:~3] OR "observational studies"[TIAB:~3]
