# Supplementary material for: Reinforcing Gaps? A Rapid Review of Innovation in Borderline Personality Disorder (BPD) Treatment
Source: Brain Sci. 2025 Jul 31;15(8):827. doi: 10.3390/brainsci15080827 (PMC12384757; doi:10.3390/brainsci15080827)
Supplement: Supplementary file 1 [file brainsci-15-00827-s001.zip › PROMPT.pdf]

PROMPT

We are conducting a scoping review on therapeutic innovations in borderline personality disorder over the past five years.

Please extract data into an Excel spreadsheet from the attached articles. For each item below, pull the exact numbers or text from the manuscript; if information is not reported, enter 'NR' ("not reported") or "None reported" as appropriate.

1. First author; Year
2. Geographic setting: Country (or main countries if multicenter)
3. Study design: RCT, quasi-experimental, pilot, prospective/retrospective observational, etc.
4. Primary objective: Exact wording from manuscript
5. Population inclusion/exclusion criteria:

o Inclusion: as listed in Methods
o Exclusion: as listed in Methods (enter "NR" if none stated)

6. Sample size (N)
7. Demographics (from baseline table):

o Mean age (± SD) (enter "NR" if missing)
o % female (enter "NR" if missing)

8. Innovation classification: Psychotherapy / Pharmacological / Digital tool / Neuromodulation / Other
9. Intervention description (expand all acronyms):

o Name
o Duration (weeks/months)
o Frequency (e.g., weekly, biweekly)
o Format (individual, group, mixed)
o Key modules/components

10. Comparator / Control: List all, with details of control condition
11. Targeted outcome domains: Check Yes/No, and if "Yes," specify details:

o Suicide mortality
o Physical morbidity/mortality
o Physical symptoms (which ones)
o BPD symptoms
o Other psychiatric disorders (which)
o Psychosocial functioning (QoL, work, relationships)
o Societal impact (stigma, caregiver burden; specify)

12. Variables used (acronyms & definitions): e.g. "BPDSI = Borderline Personality Disorder Severity Index"
13. Vulnerable populations included: Note any explicit mention of minors (< 18 yrs), elderly (> 65 yrs), LGBTQIA+, racial/ethnic minorities, migrants, etc.; enter "None reported" if not mentioned.
